# Microvascular density and hypoxia-inducible factor pathway in pancreatic endocrine tumours: negative correlation of microvascular density and VEGF expression with tumour progression

**DOI:** 10.1038/sj.bjc.6602245

**Published:** 2004-11-23

**Authors:** A Couvelard, D O'Toole, H Turley, R Leek, A Sauvanet, C Degott, P Ruszniewski, J Belghiti, A L Harris, K Gatter, F Pezzella

**Affiliations:** 1 Department of Pathology, Hôpital Beaujon, 100 Boulevard du Général Leclerc, 92110 Clichy, France; 2 Department of Gastroenterology, Hôpital Beaujon, 100 Boulevard du Général Leclerc, 92110 Clichy, France; 3 Cancer Research UK, Tumor Pathology Unit, University of Oxford, John Radcliffe Hospital, Oxford OX3 9DU, UK; 4 Department of Surgery, Hôpital Beaujon, 100 Boulevard du Général Leclerc, 92110 Clichy, France

**Keywords:** pancreas, endocrine tumours, angiogenesis, Hypoxia, HIF

## Abstract

Tumour-associated angiogenesis is partly regulated by the hypoxia-inducible factor (HIF) pathway. Endocrine tumours are highly vascularised and the molecular mechanisms of their angiogenesis are not fully delineated. The aim of this study is to evaluate angiogenesis and expression of HIF-related molecules in a series of patients with pancreatic endocrine tumours (PETs). The expression of vascular endothelial growth factor (VEGF), HIF-1*α*, HIF-2*α* and carbonic anhydrase 9 (CA9) was examined by immunohistochemistry in 45 patients with PETs and compared to microvascular density (MVD), endothelial proliferation, tumour stage and survival. Microvascular density was very high in PETs and associated with a low endothelial index of proliferation. Microvascular density was significantly higher in benign PETs than in PETs of uncertain prognosis, well-differentiated and poorly differentiated carcinomas (mean values: 535, 436, 252 and 45 vessels mm^−2^, respectively, *P*<0.0001). Well-differentiated tumours had high cytoplasmic VEGF and HIF-1*α* expression. Poorly differentiated carcinomas were associated with nuclear HIF-1*α* and membranous CA9 expression. Low MVD (*P*=0.0001) and membranous CA9 expression (*P*=0.0004) were associated with a poorer survival. Contrary to other types of cancer, PETs are highly vascularised, but poorly angiogenic tumours. As they progress, VEGF expression is lost and MVD significantly decreases. The regulation of HIF signalling appears to be specific in pancreatic endocrine tumours.

Tumorigenesis is critically dependent on the development of a vascular supply and the regulators of blood vessel growth have been shown to play a coordinated role in the progression of a variety of tumours (Bergers and Benjamin, 2003). Normal endocrine tissues and endocrine-derived tumours are characteristically highly vascular and possess a dense and specialised permeable microvascular endothelium. Endocrine cells overproduce the angiogenic peptide vascular endothelial growth factor (VEGF), which is likely to play an important role in the angiogenic process associated with endocrine tumorigenesis (Christofori *et al*, 1995; Terris *et al*, 1998; Katoh, 2003). Moreover, they also express specific endothelial cell mitogens, such as endocrine-gland-derived VEGF, (EG-VEGF) that may function in a complementary manner (LeCouter *et al*, 2001). However, the local and highly specific mechanisms acting to regulate the angiogenic process associated with endocrine tumorigenesis are poorly understood.

Hypoxia and the hypoxia-inducible factor-1 (HIF-1) pathway regulate the expression of a diverse group of genes that promote tumour growth and are involved in tissue invasion, angiogenesis, cell proliferation, glycolysis and pH regulation (Semenza, 2001; Wykoff *et al*, 2001; Harris, 2002; Maxwell and Ratcliffe, 2002; Pugh and Ratcliffe, 2003). Under normal conditions, the HIF-1*α* subunit is hydroxylated by proline hydroxylase in a reaction requiring oxygen, undergoes ubiquitination by the von Hippel-Lindau (VHL) protein and is then rapidly degraded in the proteosome. Under conditions of hypoxia in many cancers, HIF-1*α* cannot be degraded and increases in the nucleus, leading to the upregulation of many hypoxia-response proteins, such as VEGF or EG-VEGF and carbonic anhydrase 9 (CA9) (Pastorekova *et al*, 1992; Opavsky *et al*, 1996; Wykoff *et al*, 2000; Loncaster *et al*, 2001; Hui *et al*, 2002). The Hif-2*α* subunit shows properties similar to Hif-1*α*, but is mainly expressed in the stromal macrophages, and may mediate a different response to hypoxia (Talks *et al*, 2000). The HIF-1 pathway can also be activated in the VHL syndrome as a consequence of somatic VHL gene inactivation (Kaelin, 2002). Pancreatic endocrine tumours can occur both sporadically and as part of a VHL syndrome, which suggests that they may be a good model for studying the potential role of the oxygen-sensing pathway in endocrine tumours.

The aim of this study was to establish the expression pattern of VEGF, HIF-1*α*, HIF-2*α* and CA9 in a series of pancreatic endocrine tumours and to correlate the level of expression with clinicopathological charateristics, angiogenesis and survival.

## MATERIAL AND METHODS

### Patients and tissues (Table 1)

Paraffin-embedded blocks from 45 pancreatic endocrine tumours (PET) were retrieved, from 45 patients (37 patients undergoing surgery, seven patients undergoing diagnostic biopsy and one autopsy) followed at the Beaujon hospital between 1995 and 2001. Surgery consisted of splenopancreatectomy in 17 patients, pancreaticoduodenectomy in 12 and limited resection of the pancreatic body and/or neck (*n*=4) or tail (*n*=4). Biopsy specimen included five from liver metastases and two from pancreatic tumours. One block was selected for each case, containing the tumour and the peripheral nontumoral pancreas when possible in large pancreatic resections (*n*=33). The nontumoral pancreas consisted of chronic pancreatitis associated with areas of normal pancreas in 24 cases. Tissue sections (4 *μ*m) were cut and flanking sections from each tumour sample were studied for the expression of VEGF, HIF-1*α*, HIF-2*α*, CA9 and CD34.

The clinicopathological characteristics of the 45 cases are summarised in Table 1. Survival data were available for 43 out of 45 patients. One patient who died within 1 month after operation was excluded to avoid bias from peri-operative death. The median follow-up of surviving patients at the time of analysis was 32 months (range 2–86 months). The following histopathological and clinical data were recorded for surgical or autopsy material: age, sex, functional status of tumours (hormonal syndrome (insulinoma, glucagonoma, somatostatinoma, VIPoma) or not), VHL disease and type 1 multiple endocrine neoplasia (MEN1) syndrome. Tumours were classified in four groups according to the WHO 2000 criteria (benign well-differentiated endocrine tumours, reported as WHO-1, well-differentiated endocrine tumours of uncertain behaviour, reported as WHO-2, well-differentiated endocrine carcinomas, reported as WHO-3, and poorly differentiated endocrine carcinomas, reported as WHO-4) (Solcia *et al*, 2000). Most of the WHO-4 cases corresponded to biopsy specimen because such cases which require chimiotherapy are not surgically resected. VHL cases corresponded to WHO-1 (*n*=1), WHO-2 (*n*=3) or WHO-3 (*n*=4) tumours. In addition, the size of the tumour, presence of necrosis, presence of a fibrotic focus, number of mitoses (per 10 high power field), percentage of cell nuclei stained for Ki-67 (MIB-1 antibody), vascular embolism (not evaluated in the biopsy samples), lymph node metastasis and liver metastasis were also recorded. Tumours with ⩽5 mitoses per 10 high-power field or ⩽5% Ki-67-positive cells were considered at a low mitotic rate or low proliferative index, respectively. In accordance with previously reported studies, both poorly differentiated tumours and tumours with high proliferative index were associated with poorer survival in our series (*P*<0.0001) (Gentil Perret *et al*, 1998; Madeira *et al*, 1998).

### Immunohistochemistry

#### Antibodies and immunohistochemical techniques

The VEGF, HIF-1*α*, HIF-2*α*, CA9, Ki-67 proteins and CD34 antigen were detected using the following murine monoclonal antibodies: VG1 (that detects the 121, 165 and 189 VEGF isoforms), ESEE 122, Ep 190b, M75, MIB-1 (Dako) and QBEND10 (Dako), respectively (Turley *et al*, 1998; Talks *et al*, 2000; Hui *et al*, 2002). The methodology used is as described previously (Turley *et al*, 1998; Hui *et al*, 2002). Briefly, sections were first placed in a 60°C oven for 10 min before deparaffinisation in Citroclear twice for 12 min and rehydratation. Antigen retrieval consisted of pressure cooking for 3 min in Tris-EDTA (pH 9) for VEGF, Ki-67 and CD34, and 60°C waterbath overnight in 1 mM Tris-EDTA (pH 9) for HIF-1*α* and HIF-2*α*. Permeabilisation was performed with 0.2% triton X-100 for 10 min for HIF-1*α* and HIF-2*α*. Endogenous peroxidase was then quenched with Dako peroxidase block solution applied for 5 min. The primary antibody was applied, rinsed in PBS and then the secondary polymer from the Envision HRP kit (Dako) was applied for 30 min. After the slides were washed in PBS, the colour was developed by a 5-min incubation with 3,3′-diaminobenzidine solution (Dako). Sections were counterstained with haematoxylin and mounted. PBS was substituted for primary antibody as the negative control. Positive controls consisted of serum in blood vessel lumen (VEGF), HIF-1*α* transfected COS-1 cells (HIF-1*α*), known positive tumour (HIF-2*α*) and clear cell renal carcinoma (CA9).

Immunohistochemical double staining of CD34 and Ki-67 was performed to detect proliferating endothelial cells. The immunohistochemical reaction against Ki-67 was revealed in brown with 3,3′-diaminobenzidine and was followed by the immunohistochemical reaction against CD34 revealed in red with amino ethyl carbazole. The brown nuclei of proliferating endothelial cells are superimposed on the red colour of the cytoplasms.

#### Scoring methods

All slides were evaluated independently by two investigators (AC and KG) who were blinded to the patients' clinical data. The differences in evaluation between the two observers were resolved at a conference microscopy. The specimens were scanned at a low optical power (× 40) to study the tissue distribution of staining and at a high optical power (× 250) to study the cellular staining patterns. The percentage of cells with positive reactivity was scored. The pattern of expression (cytoplasmic, membranous or nuclear) and the intensity (negative scored as 0, weak scored as 1, moderate scored as 2 and strong scored as 3) were noted. A cytoplasmic score was calculated by multiplying the percentage of cytoplasmic stained cells by their staining intensity. For HIF-2*α* the presence or absence of positively stained stroma was noted. The staining of the stroma was not evaluated in biopsy cases because of the small size of these tissue samples.

#### Microvessel counting

Two areas of high vascularisation were chosen for microvessel counting at a low optical power (× 10 objective) after CD34 staining. The final microvessel density (MVD) was the mean value of three appraised high power fields (× 25 objective, Leitz, field area 0.442 mm^2^) in each area of high vascularisation (total area: 2.65 mm^2^). The vessel counts were very homogeneous in all tumours and six fields were sufficient to obtain reproducible results. In biopsy samples, the entire biopsy was evaluated for microvessel counting. Vessels with a clearly defined lumen or well-defined linear vessel shape were taken into account for counting. Tumours with <200 microvessels mm^−2^ were considered as low microvessel density, whereas those with >200 microvessels mm^−2^ were considered as high microvessel density.

#### Proliferating endothelial cells counting

The percentage of proliferating endothelial cells was determined in areas of high vascularisation using a × 40 objective. In all, 300 endothelial cells were recorded as positive or negative for Ki-67 in each tumour. In biopsy samples, the entire biopsy was evaluated for proliferating endothelial cells counting.

### Statistical analysis

Fisher's exact tests examined relationships between categorical tumour variables. Mann–Whitney nonparametric tests were utilised to compare categorical with continuous tumour variables where the number of categories was two; where the number of categories was greater than two, Kruskall–Wallis nonparametric tests were used instead. Spearman rank correlations were used to investigate relationships between continuous patient and tumour variables. *P*-values of <0.05 were considered significant. Survival rates were calculated from the time of resection until the end of follow-up period (July 2003) and curves were plotted using the method of Kaplan and Meier. The log-rank test was used to evaluate differences between life tables. These analyses were performed using Statview 4.5 statistical analysis software (Abacus Concepts Inc., CA, USA).

## RESULTS

### VEGF expression

In nontumoral pancreas, VEGF was detected in islets with a strong intensity in all cases. Normal or reactive hyperplastic ducts and acini were persistently negative.

Tumour cells had positive cytoplasmic staining in 33 (73%) patients (weak intensity in 18 cases, moderate intensity in 10 cases and strong intensity in four). The VEGF score ranged from 0 to 210 (mean 55; median 30).

**Figure 1:**
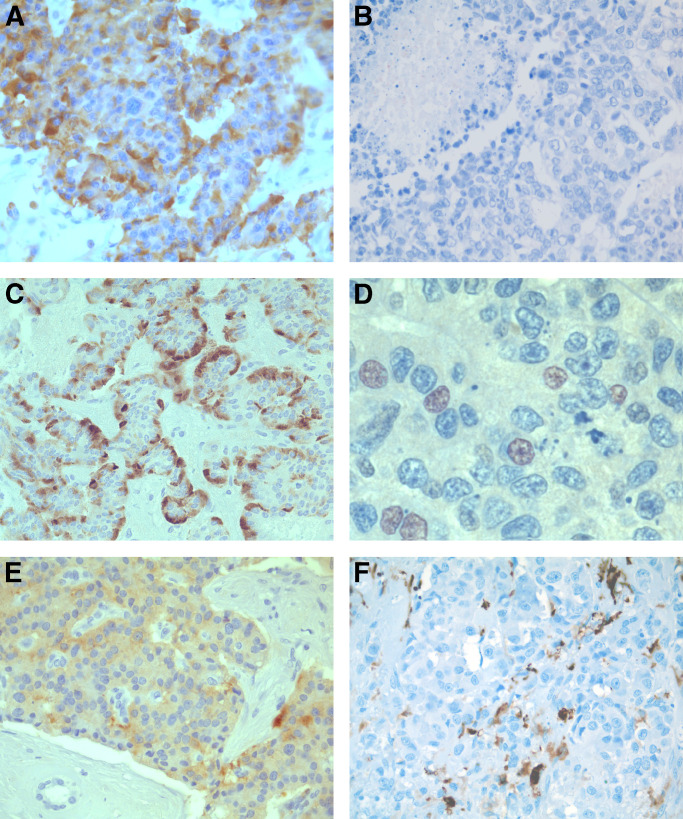
Immunohistochemical expression of VEGF, HIF-1*α* and HIF-2*α* by WHO-stage 1 (**A, C, E** and **F**) and WHO-stage 4 (**B, D**) PETs. (**A, B**) VEGF is highly expressed in a WHO stage-1 PET (**A**) and negative in a WHO-stage 4 PET (**B**). (**C, D**) HIF-1*α* cytoplasmic expression is strong in a WHO-stage 1 PET (**C**). HIF-1*α* nuclear expression is detected in a WHO-stage 4 PET (**D**). (**E, F**) HIF-2*α* cytoplasmic (**E**) and stromal (**F**) expression is detected in a WHO-stage 1 PET. Immunoperoxidase and haematoxylin nuclear counterstaining; original magnifications: **A**, × 250; **B**, × 250; **C**, × 150; **D**, × 500; **E**, × 200; **F**, × 250.

**Figure 2:**
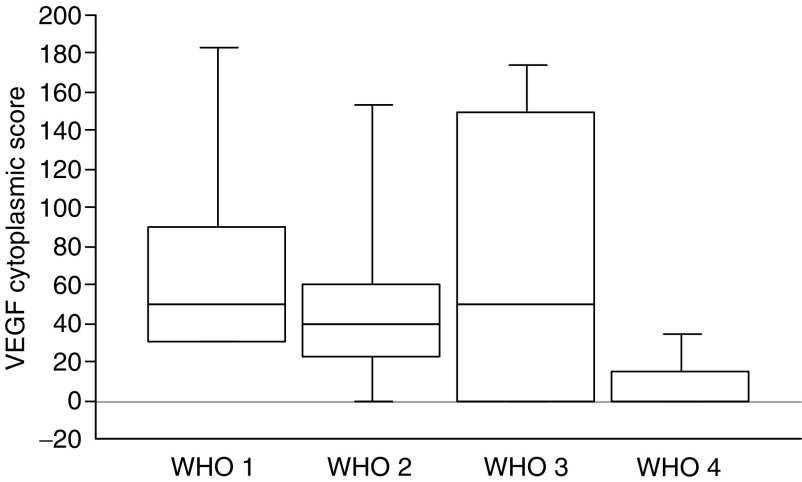
Box plot of VEGF cytoplasmic score stratified according to the WHO classification. Differences between groups were statistically significant (*P*=0.03).

VEGF expression correlated negatively with WHO disease stage (Figures 1A, B and 2). Five of eight patients (62%) with negative staining for VEGF had WHO-stage 4, five out of 18 (28%) WHO-stage 3 and two out of 19 (11%) WHO-stage 1–2. The VEGF score was significantly higher in tumours with a low index of proliferation or mitosis count, no necrosis, no fibrotic focus and a high microvascular density (although this did not reach significant levels; *P*=0.06). No difference in VEGF expression was observed in endocrine tumours associated with VHL or not (see values in Table 2). There was no association between VEGF expression and survival.

### HIF-1*α* expression

In nontumoral pancreas, HIF-1*α* was detected in islets with a high cytoplasmic intensity in all cases and was expressed by nerves and neurons.

The majority of endocrine tumours expressed high levels of cytoplasmic HIF-1*α*. Only four cases (9%) showed no HIF-1*α* cytoplasmic staining. Percentage values varied from 0 to 100% of tumour cells (mean cytoplasmic score 167; median 180). The intensity of cytoplasmic staining was moderate or strong in 35 cases. Nuclear staining was detected in 31 out of 45 (69%) of the cases (percentage values ranged from 0 to 50%; mean 9.3%; median 5%).

**Figure 3:**
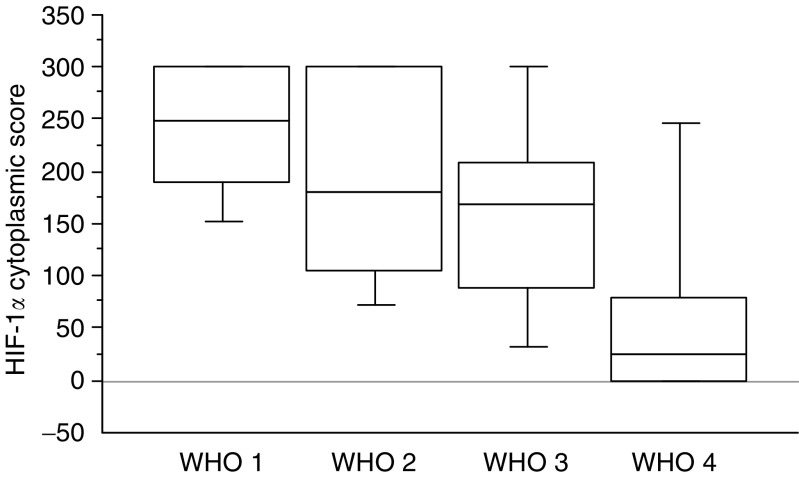
Box plot of HIF-1*α* cytoplasmic score stratified according to the WHO classification. Differences between groups were statistically significant (*P*=0.008).

HIF-1*α* cytoplasmic expression was significantly higher in well-differentiated benign tumours (Figure 1C) than in tumours of uncertain behaviour or carcinomas (see values in Figure 3). The HIF-1*α* cytoplasmic score was significantly lower in poorly differentiated tumours, tumours of large size, with high mitosis count, necrosis, lymph node metastasis, low MVD or VHL disease (see values in Table 2).

**Figure 4:**
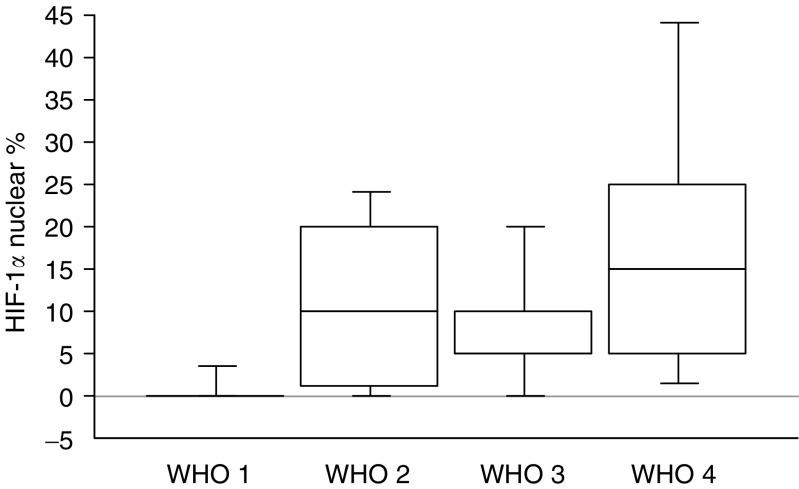
Box plot of HIF-1*α* nuclear expression stratified according to the WHO classification. Differences between groups were statistically significant (*P*=0.001).

HIF-1*α* nuclear expression was significantly higher in carcinomas (Figure 1D) than in tumours of uncertain behaviour and in benign tumours (Figure 4). Presence of HIF-1*α* nuclear expression was significantly associated with poor tumour differentiation, large tumour size, higher mitosis count or index of proliferation, presence of necrosis, of a fibrotic focus, of liver metastasis, low MVD and VHL disease (mean values in Table 2). Presence of HIF-1*α* nuclear expression was associated with shorter survival, although this was not statistically significant (*P*=0.06).

### HIF-2*α* expression

Nontumoral pancreatic islets showed a weak cytoplasmic HIF-2*α* staining. Tumour cells were found to have cytoplasmic staining (Figure 1E) in 13 cases (29%) and nuclear stainings in 13 (29%) cases. Both stainings were not statistically associated. Percentage staining of cytoplasm varied from 5 to 80% of PET cells (mean cytoplasmic score 13; median 0). Nuclear staining was weak (range 1–30%; mean 1.5%; median 0).

Stromal HIF-2*α* expression was observed in 12 out of 38 (32%) of cases, detected in the cytoplasm of tumour-associated macrophages which were present in moderate to large numbers (Figure 1F). None or few positive macrophages were considered as negative.

There was no significant association of HIF-2*α* staining in tumour cells or in macrophages with histopathological variables, MVD, VHL disease or survival.

### CA9 expression

In nontumoral pancreatic tissue, CA9 was detected in normal or reactive hyperplastic ducts in 22 cases and in acini in 12 cases. Islets were negative. Expression of CA9 in the normal pancreas was stronger in areas of pancreatitis close to invading tumours.

**Figure 5:**
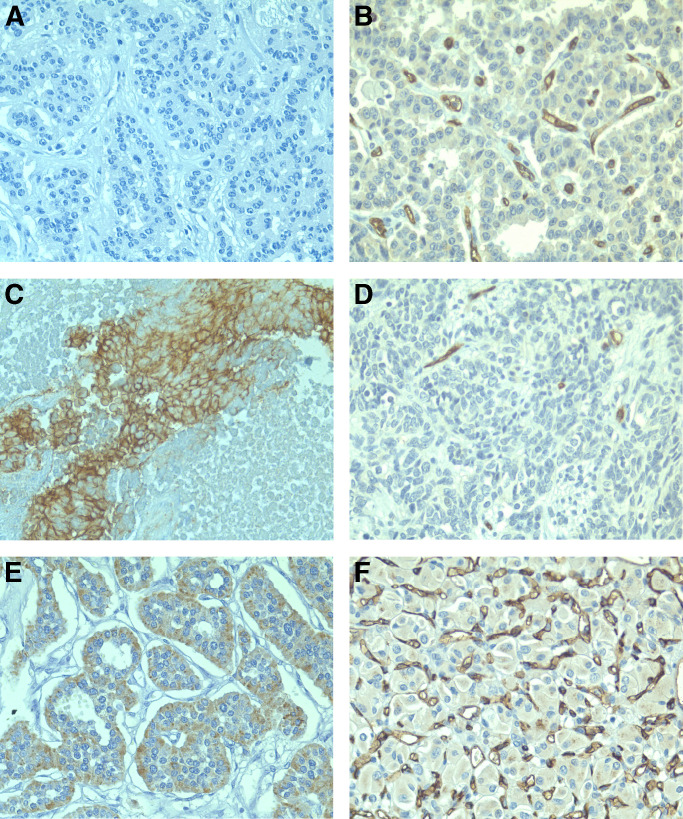
Immunohistochemical expression of CA9 and CD34 by WHO-stage 1 (**A, B**) and WHO-stage 4 (**C, D**) pancreatic endocrine tumours and by VHL cases (**E, F**). (**A, C, E**) CA9 is not detected in most well-differentiated PETs (**A**). Its expression is strong and membranous around areas of necrosis in WHO-stage 4 PETs (**C**) and cytoplasmic diffuse in VHL cases (**E**). (**B, D, F**) CD34+ capillaries are numerous in WHO-stage 1 PETs (**B**) and scattered in WHO-stage 4 PETs (**D**). Microvascular density is very high in VHL cases (**F**). Immunoperoxidase and haematoxylin nuclear counterstaining; original magnifications: **A**, × 150; **B**, × 250; **C**, × 200; **D**, × 150; **E**, × 200; **F**, × 250.

CA9 expression was cytoplasmic (of weak or moderate intensity) in nine tumours or membranous (of moderate or strong intensity) in 12 tumours. No CA9 staining was seen in 25 out of 45 (53%) of tumours (Figure 5A). Percentage values of membranous or cytoplasmic staining varied from 5 to 60% (mean score 22; median 0) and from 5 to 80% (mean score 15; median 0) of tumour cells, respectively. Fibroblasts were positive in only one case, in the tumoral stroma adjacent to a fibrotic area, in a case containing membranous CA9+ tumour cell areas.

**Figure 6:**
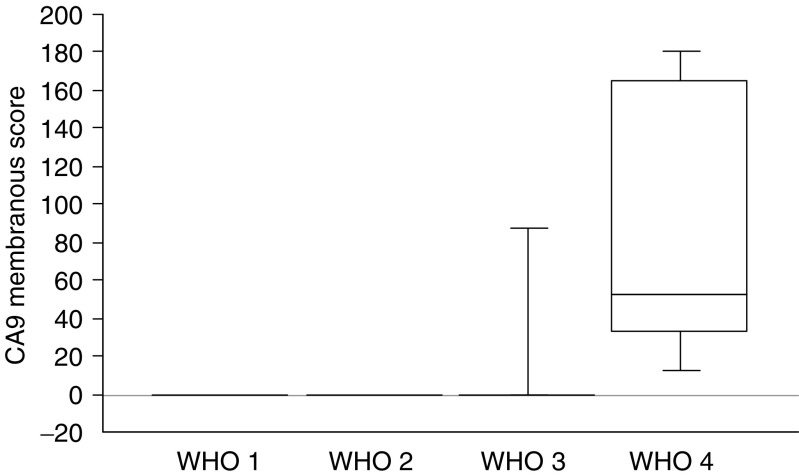
Box plot of CA9 membranous expression stratified according to the WHO classification. Differences between groups were statistically significant (*P*=0.0001).

**Figure 7:**
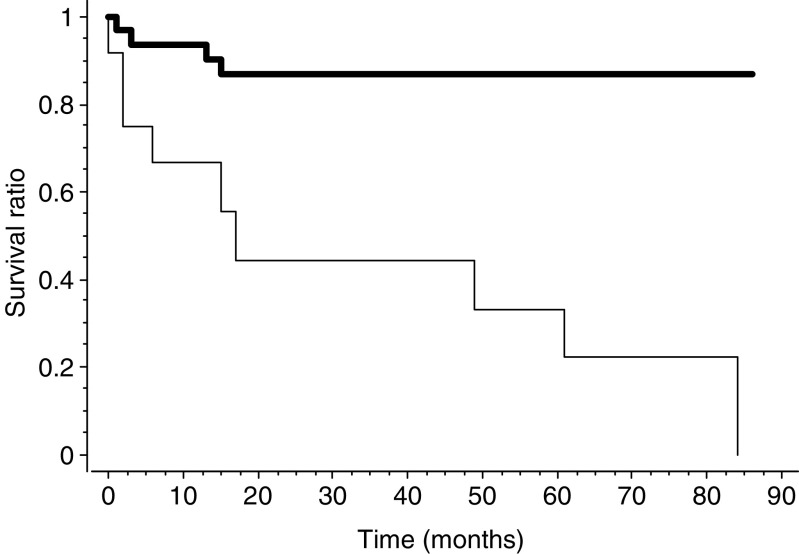
Survival curves according to Kaplan–Meier for 43 of the 45 patients of the study group according to CA9 membranous expression (thin line) or absence of expression (bold line); *P*=0.0004.

CA9 membranous expression (Figure 5C) was significantly higher in WHO-stage 4 or poorly differentiated tumours (see values in Figure 6). Out of 12 PETs with CA9 membranous staining, 10 contained necrosis either extensive or focal and CA9 expression was confined around areas of necrosis in seven of those 10 cases. Membranous expression of CA9 was significantly higher in large tumours, in tumours with the presence of a fibrotic focus, a high mitotic count or proliferation index, liver metastases, low MVD and shorter survival (*P*=0.0004; Figure 7).

CA9 cytoplasmic expression (Figure 5E) was significantly higher in tumours associated with VHL disease (values in Table 2) and in tumours with a greater microvascular density. Cytoplasmic staining was detected in eight out of eight VHL patients, whereas 36 out of 37 non-VHL patients were negative.

### Microvessel counting

**Figure 8:**
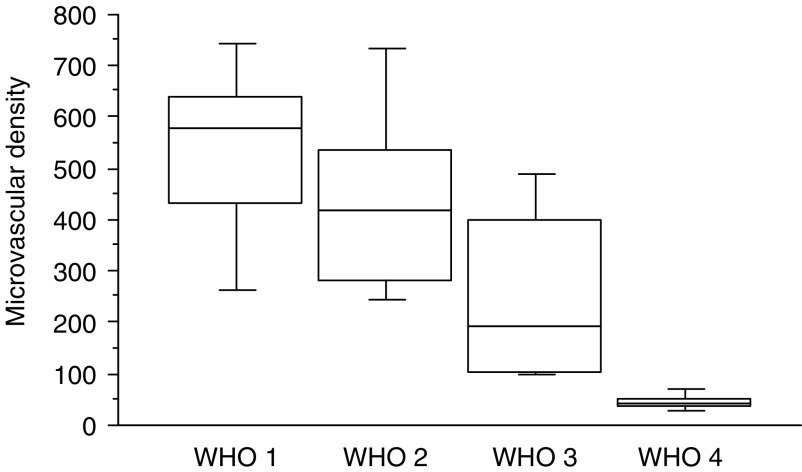
Box plot of MVD recorded in hotspots stratified according to the WHO classification. Differences between groups were statistically significant (*P*=0.0001).

**Figure 9:**
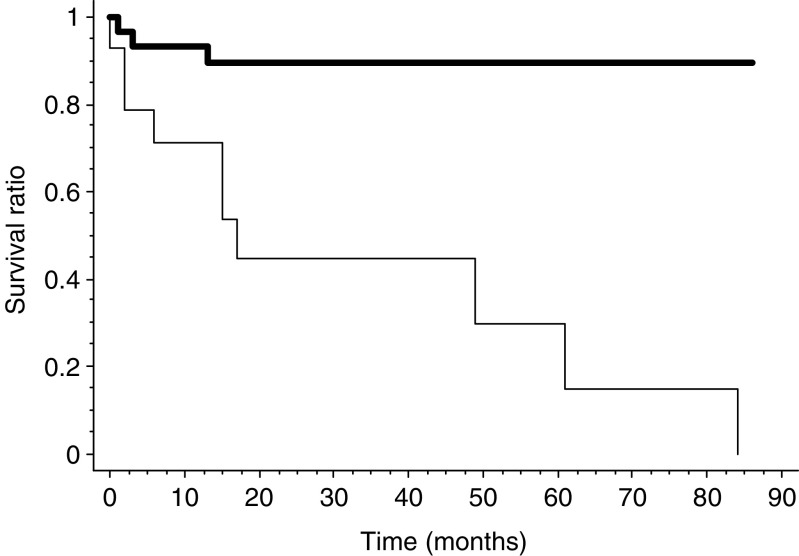
Survival curves according to Kaplan–Meier for 43 of the 45 patients of the study group according to MVD <200 mm^−2^ (thin line) or >200 mm^−2^ (bold line); *P*=0.0001.

The microvessel density ranged from 26 to 792 vessels mm^−2^ (mean 311; median 276). The mean values, (see Figure 8), decreased with disease progression according to WHO classification (Figure 5B and D). All well-differentiated tumours, both benign and of uncertain behaviour, had >200 vessels mm^−2^ (in contrast to the nine out of 18 well-differentiated carcinomas and eight out of eight poorly differentiated carcinomas, which had counts of less than 200 vessels mm^−2^). The MVD was higher in large tumours, in tumours with necrosis, a fibrotic focus, a high mitotic count or MIB1 index and presence of liver metastasis. A high MVD was also associated with VHL cases (Figure 5F and values in Table 2). Low tumour MVD (<200 vessels mm^−2^) was significantly associated with shorter survival (*P*=0.001; Figure 9).

### Proliferating endothelial cells counting

There was a mean endothelial proliferation index of 3.7% (range 0–9.6%; median 3.8%). Endothelial proliferation did not differ significantly between the different groups of the WHO classification.

### Co-expression of markers

Tumour HIF-1*α* nuclear expression was significantly correlated with that of CA9 membranous expression and low MVD. High cytoplasmic HIF-1*α* expression was correlated significantly with VEGF expression and high MVD.

## DISCUSSION

Currently, standard histology coupled with evaluation of mitotic rates and proliferation index are essential in helping to predict the biological behaviour of endocrine tumours and select appropriate treatment strategies as a function of WHO disease stage. Such methods are not always reliable and many tumours fall in-between tumour stage, indicating that better markers of tumour aggressiveness are required. The fact that endocrine tumours are highly vascular in nature renders analysis of angiogenesis as a prognostic factor highly pertinent. To date, few data evaluating angiogenesis in the progression of endocrine neoplasms are available and mostly applies to pituitary tumours where results remain equivocal (Erroi *et al*, 1986; Jugenburg *et al*, 1995; Sasano *et al*, 1998; Turner *et al*, 2000a, 2000b, 2000c; Bernini *et al*, 2002). A high intratumoral MVD was demonstrated in our study, much greater than those reported in any type of tumours (Goulding *et al*, 1995; Fox, 1997; Prall *et al*, 2003). However, we found a wide range of MVD values according to the malignant potential, undifferentiated carcinomas showing a 10 times lower MVD than benign tumours. A negative correlation has previously been described between MVD and advanced pituitary adenomas and carcinomas (Erroi *et al*, 1986; Jugenburg *et al*, 1995; Bernini *et al*, 2002), and a recent study demonstrates comparable results in PETs (Marion-Audibert *et al*, 2003). In our series, a high MVD was found to correlate with previously established markers of good prognosis (small tumour size, well-differentiated tumours, low mitotic count and low proliferation index) (Solcia *et al*, 2000; Hochwald *et al*, 2002). Moreover, patients with low MVDs had a significantly shorter survival (*P*=0.0001) and a higher risk of metastatic dissemination. These observations appear to be tumour-specific and contrast with those seen previously in most other epithelial cancers (Weidner *et al*, 1992; Maeda *et al*, 1995; Weidner, 1995, 1998; Pezzella *et al*, 1997; Ellis *et al*, 1998; Eberhard *et al*, 2000). The specific vascular profile observed in PETs may help to predict their prognosis. In fact, prediction of the biological behaviour of PETs is difficult by histological criteria alone and patients with metastatic disease may have a prolonged survival. MVD may help in predicting long-term prognosis in these patients. As MVD has also been shown to be correlated with contrast enhancement on computed tomography in PET (unpublished personal data), use of such a marker would not be limited to post-operative scenarios.

We demonstrate in this study a specific pattern of expression of VEGF, HIF-1*α* and HIF-2*α* in pancreatic endocrine tumours. VEGF is upregulated in many human tumours and its expression has usually been correlated with a high microvascular density and poor clinical outcome (Itakura *et al*, 1997; Seo *et al*, 2000; Hui *et al*, 2002; Niedergethmann *et al*, 2002). In the present study, VEGF expression detected in 78% of PETs, mainly in highly vascularised benign cases, is in accordance with previous results (Terris *et al*, 1998; La Rosa *et al*, 2003). An interesting finding is that VEGF expression was significantly lower in undifferentiated poorly vascularised cases.

HIF-1*α* and HIF-2*α* are known to mediate the induction of the HIF-regulated genes. Their nuclear expression has been demonstrated in several tumours and is associated with an aggressive phenotype (Zhong *et al*, 1999; Birner *et al*, 2000; Giatromanolaki *et al*, 2001). Expression of such proteins in perinecrotic areas supports evidence for microenvironmental mechanisms of activation secondary to hypoxia (Talks *et al*, 2000). To date, their expression has never been described in endocrine tumours. We have demonstrated a cytoplasmic distribution of HIF-1*α* in 91% of PETs and HIF-2*α* in 29%, which has rarely been reported in other human tumours (Zhong *et al*, 1999; Talks *et al*, 2000; Zagzag *et al*, 2000; Giatromanolaki *et al*, 2001; Hui *et al*, 2002). This expression appears not to be related to a local hypoxic process, since there is a high number of microvessels in these tumours. Moreover, it is not known whether these cytoplasmic molecules are active or not. We also demonstrated, as for VEGF, the cytoplasmic expression of HIF-1*α* and HIF-2*α* in normal pancreatic islets. This specific phenotype, which persists in benign PETs, is progressively lost with disease progression. Endocrine blood vessels are numerous and stabilised in normal endocrine tissue and probably retain these properties in well-differentiated tumours. This is supported by the low index of proliferation of endothelial cells, which suggests that despite a high MVD there is a low neoangiogenesis in these tumours. As suggested by the observation of low MVD in advanced carcinomas, vessels progressively regress during tumour progression. This is consistent with recently described animal models showing loss of vessel differentiation and a decrease in vessel density in pancreatic endocrine tumours as compared to the normal islets (Ryschich *et al*, 2002). Expression of antiangiogenic factors probably plays a role in this phenomenon (Holash *et al*, 1999; Bergers and Benjamin, 2003; Carmeliet, 2003). In advanced PETs, low MVD associated with an increase in tumour-cell proliferation may, beyond the limit of oxygen diffusion, lead to hypoxia and activation of CA9. This pattern of expression resembles that of most other carcinomas with strong and membranous positivity for CA9 at the periphery of areas of necrosis (Zhong *et al*, 1999; Wykoff *et al*, 2000; Loncaster *et al*, 2001; Hui *et al*, 2002). CA9 expression is consistent with the upregulation of HIF-1*α* nuclear expression in poorly differentiated tumours, recalling the situation observed in most carcinomas studied so far. The value of membranous CA9 was underlined by its strong associations with factors of poor outcome (tumour size and differentiation), but especially with a shorter survival (*P*=0.0004). A diffuse cytoplasmic CA9 pattern of expression as a specific feature of VHL cases was observed in our series, differing from the strong membranous staining that we found in undifferentiated tumours. In most tumours, CA9 is a transmembrane enzyme with an extracellular active site, linked to aggressive tumour behaviour (Opavsky *et al*, 1996; Wykoff *et al*, 2000; Loncaster *et al*, 2001; Hui *et al*, 2002; Turner *et al*, 2002). The role of cytoplasmic CA9, if any, is probably different in PETs. It does not appear to be hypoxia-related, as it is correlated to VHL disease in which PETs possess a significantly higher MVD but is likely to be related to loss of VHL function. In cases of VHL disease, hypoxia-inducible gene expression is constitutively upregulated (Kaelin, 2002). This is consistent with the frequency of nuclear HIF-1*α* that we found in VHL-related PETs. In renal cell carcinomas, VHL inactivation occurs in most sporadic cases, resulting in an accumulation of CA9, HIF-1*α* and HIF-2*α* (Ivanov *et al*, 1998; Turner *et al*, 2002). Our results, showing no CA9 accumulation in well-differentiated, non-VHL tumours, are consistent with the fact that VHL mutations have only been reported in the initial progression of a small proportion of PETs (Chung *et al*, 1997; Moore *et al*, 2001; Gumbs *et al*, 2002). Only one PET expressed weak levels of cytoplasmic CA9 without any clinical symptoms of VHL disease in our series. It could be hypothesised that this case may represent a sporadic VHL mutation.

In conclusion, this study shows that as PETs progress there is loss of the baseline VEGF expression and decresead MVD. There is a partial switch to hypoxia pathways in malignant undifferentiated tumours, but with an overall low angiogenic activity. Recent experimental observations have shown the markedly different effects of HIF on tumour biology in different tissues (Blouw *et al*, 2003). This is the first clinical study to demonstrate such effects in the pancreatic endocrine tumour pathway, confirming the existence of tissue and tumour-specific angiogenesis and pathway of regulation of HIF signalling.

## Figures and Tables

**Table 1:** Patients demographics and main pathological features

**Characteristics**	**No of patients (*n*=45)**
*Age (years)*	
<40	12
>40	33
	
*Sex*	
Male	22
Female	33
	
*VHL disease*	
VHL+	8
VHL−	37
	
*Functional Syndrome*	
Present	7
Absent	38
	
*Size (mm)*	
<30	25
>30	20
	
*Classification according to WHO 2000*	
WHO-stage 1=benign endocrine tumours	8
WHO-stage 2=endocrine tumours of incertain behaviour	11
WHO-stage 3=well-differentiated endocrine carcinomas	18
WHO-stage 4=poorly differentiated endocrine carcinomas	8
	
*Necrosis*	
N0	33
N1	12
	
*Ki-67 or mitoses*	
⩽5	18
>5	17
	
*Lymph node status*	
N0	27
N1	18
	
*Liver metastasis*	
M0	30
M1	15
	
*Duration of follow-up (months)*	
Median	32
Range	2–86
Lost to follow-up	2
Death	11

**Table 2:** Cytoplasmic and nuclear expression of VEGF, HIF-1*α*, HIF-2*α*, CA9 and values of microvessel density according to VHL status in well-differentiated tumours (*n*=37)

		**VHL+ (*n*=8)**	**VHL− (*n*=29)**	***P***
VEGF	Mean cytoplasmic score±s.d.	60±55	66±64	NS
				
HIF-1*α*	Mean cytoplasmic score±s.d.	95±70	215±83	0.03
	Mean nuclear %±s.d.	12±9.4	6.3±7.7	0.04
				
HIF-2*α*	Mean cytoplasmic score±s.d.	10±20	14±28	NS
	Mean nuclear %±s.d.	1.4±1.8	2±5.5	NS
				
CA9	Mean cytoplasmic score±s.d.	73±50	2.7±14.6	0.0001
				
CD34	Microvessel density (vessels mm^−2^)	563±189	215±82	0.02

VHL+: von Hippel-Lindau disease cases; VHL−: cases without von Hippel-Lindau disease; s.d.: standard deviation; NS: nonsignificant.
